# On-line monitoring of methane in sewer air

**DOI:** 10.1038/srep06637

**Published:** 2014-10-16

**Authors:** Yiwen Liu, Keshab R. Sharma, Sudhir Murthy, Ian Johnson, Ted Evans, Zhiguo Yuan

**Affiliations:** 1 Advanced Water Management Centre, The University of Queensland, QLD, Australia; 2 District of Columbia Water and Sewer Authority, Washington DC 20032, USA; 3 Gold Coast City Council, QLD, Australia; 4 Water Corporation, WA, Australia

## Abstract

Methane is a highly potent greenhouse gas and contributes significantly to climate change. Recent studies have shown significant methane production in sewers. The studies conducted so far have relied on manual sampling followed by off-line laboratory-based chromatography analysis. These methods are labor-intensive when measuring methane emissions from a large number of sewers, and do not capture the dynamic variations in methane production. In this study, we investigated the suitability of infrared spectroscopy-based on-line methane sensors for measuring methane in humid and condensing sewer air. Two such sensors were comprehensively tested in the laboratory. Both sensors displayed high linearity (R^2^ > 0.999), with a detection limit of 0.023% and 0.110% by volume, respectively. Both sensors were robust against ambient temperature variations in the range of 5 to 35°C. While one sensor was robust against humidity variations, the other was found to be significantly affected by humidity. However, the problem was solved by equipping the sensor with a heating unit to increase the sensor surface temperature to 35°C. Field studies at three sites confirmed the performance and accuracy of the sensors when applied to actual sewer conditions, and revealed substantial and highly dynamic methane concentrations in sewer air.

Methane (CH_4_) is a highly potent greenhouse gas and contributes significantly to climate change^1^,^2^,^3^. It displays a Lower Explosive Limit (LEL) of approximately 5% by volume (vol), and thus poses a serious safety concern^4^. Thus, water utilities are committed to reducing methane emission from wastewater systems, namely sewer networks and wastewater treatment plants (WWTPs). Recently, significant progress has been made in quantifying and mitigating methane emission from WWTPs^5^,^6^. In comparison, little work has been done regarding the understanding of methane emission from sewer systems. The knowledge gap has led the Intergovernmental Panel on Climate Change (IPCC) to conclude that, “…wastewater in closed underground sewers is not believed to be a significant source of methane”^1^. However, this has been proven to be untrue by several recent studies. Dissolved methane concentrations of 5–25 mg/L were measured at several rising main sewers^7^,^8^. Corroborating the liquid phase data, the gas phase methane concentrations of up to 50,000 ppmv, i.e. 5% vol, were detected in the air of a gravity sewer^9^. In contrast, the current atmospheric methane concentration is 1.8 ppmv^9^. In a US study, gas phase methane concentrations of 500–900 ppmv, i.e. 0.05–0.09% vol, were detected at the discharge of a 5.3 km rising main with a diameter of 406 mm, yielding a CH_4_ emission of 7.44 kg/d^10^. These data confirmed significant methane production and emission from sewers, which is currently not accounted for.

Sewer systems are highly dynamic^11^. Sewage flows vary substantially over time, leading to fluctuating wastewater hydraulic retention time (HRT) in sewers. In addition, in rising main sewers, pumps are frequently turned on and off resulting in intermittent flow, which further adds to sewer dynamics. Similar to the dynamics already observed for hydrogen sulfide production in sewers^11^, CH_4_ concentrations in both the liquid and gas phases are also expected to fluctuate. Therefore, continuous monitoring of CH_4_ concentration is important for the accurate quantification and overall understanding of CH_4_ production and emission from sewers. However, manual sampling for off-line chromatographic (GC) analysis has been the primary method for CH_4_ measurement from sewers^7^,^8^,^10^. It is difficult to capture the expected fluctuation in CH_4_ concentration with this method; therefore it imposes a serious limitation on accurate quantification. In addition, methane emission data is expected to vary from site to site^7^, and manual sampling is not feasible for long-term quantification of methane concentrations over a large number of sampling sites along extensive sewer networks.

On-line sensors for continuous CH_4 _measurement potentially provide a solution to the aforementioned problems. Instruments that are able to measure CH_4_ on-line are available and have been widely used in combination with detecting systems such as infrared spectroscopy, photoacoustic spectrometry, and pellistors or metal-oxide semiconductors^12^,^13^. Photoacoustic detection is based on the photoacoustic effect in which energy from a radiation source is first converted to a sound and then to an electrical signal; this is a developing technology used for methane measurement and there are few products on the market. Pellistors are calorimetric flammable gas sensors, which detect a temperature variation when a heated catalytic element is exposed to a mixture of combustible gases. Pellistors are not expected to be applicable in sewer conditions due to the known toxic effect of hydrogen sulfide on electrodes^14^. Metal-oxide semiconductors will vary in conductance or resistance in response to the presence of different gases. The major limitation is that a semiconductor can respond to any gas that can be oxidized and hence is not specific to a particular gas^15^. Infrared (IR) spectroscopy is based on the principle of measuring an absorption line unique to the detected gas, with significant sensitivity and selectivity. Although alkane such as propane, pentane, butane and hexane can interfere with CH_4_ measurement via IR spectroscopy, these hydrocarbons are not expected to exist in sewers in significant amounts unless directly discharged^9^. Therefore, out of the methods described, IR spectroscopy is likely the most promising method for online CH_4_ measurement in sewer conditions^9^.

Gas sensors with IR spectroscopy technology are mainly used as safety devices for detecting flammable gases such as CH_4_ in underground mining and petrochemical industries^16^. Almost all commercial IR sensors are of the non-dispersive type, which uses discrete optical band-pass filters. Even though it is claimed that these sensors are applicable to methane detection in wastewater treatment facilities, sensor performance has not been fully studied and there have been no reports in the scientific literature evaluating the suitability of these sensors for on-line methane measurement in sewers.

Both temperature and humidity can affect IR sensor performance. An IR detector is essentially a temperature sensor and is, therefore, potentially sensitive to changes in the temperature. The temperature in sewers varies between day and night as well as with seasons, which may influence the sensor performance. Humidity is often a major interference with infrared systems^17^. Water vapour has a significant absorption spectrum that has peaks similar to CH_4_. Therefore, water vapour could interfere with CH_4_ signals and cause false readings due to potential overlap in spectrum. Water vapour can also condense on the optics or in the light path and cause the beam to be deflected or diffracted so that an erroneous reading or instrument failure can occur. The relative humidity in sewers is usually above 90% and sometimes it can be fully condensing^18^, and this may have an adverse effect on IR detection of CH_4_. The performance of IR sensors in humid sewers is unknown and requires evaluation before wide application to the sewer environment.

This paper provides a comprehensive evaluation of IR methane sensors for in-sewer application. In the laboratory study, linearity, detection limit, reproducibility, and effects of environmental conditions such as humidity and temperature on two types of IR methane sensors were evaluated. After that, field validation was performed to examine sensor long-term performance and accuracy when applied to actual sewer conditions. This paper provides scientific evidence to support the wide application of IR methane sensors for methane measurement in sewers.

## Results

### Linearity, limit of detection and reproducibility

**Figure 1:**
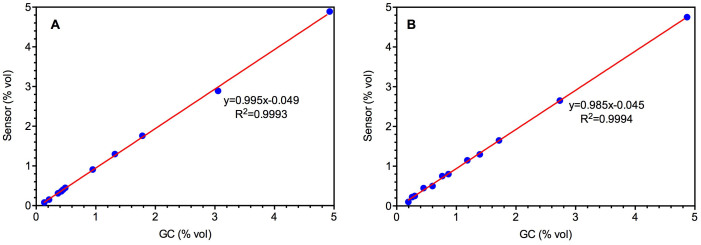
Calibration of Sensor I (A) and Sensor II (B) at 54% RH and 23 ± 1°C.

Two IR CH_4 _sensors, i.e. one portable gas detector (OdaLog 7000 IR, operated with battery, named as Sensor I in this paper) and one fixed gas detector (GasTech S-Guard IR, operated with external power supply, named as Sensor II in this paper), were selected for testing. According to the data reported in a study conducted on 14 manholes in a sewer line in Melbourne^9^, gas phase methane concentrations usually vary from 1000 to 50000 ppm (or 0.10–5.00% vol), which was chosen as the basis for the calibration range. Fig. 1 presents laboratory results from the linearity tests, by showing sensor measurements against the off-line GC measurements. The response of both sensors to the change in methane concentration was linear up to 5% vol (the highest concentration tested in this study) with the R^2^ values above 0.999 for both sensors. The ratio between the sensor and the GC readings was 0.995 for Sensor I and 0.985 for Sensor II. Both ratios are close to 1, indicating that the factory calibration can be used for CH_4_ measurement under conditions applied in these tests, and no further calibration is required. Also, the observed intercept on the y-axis for both sensors was about −0.05, which is close to zero. These results indicate both sensors are able to measure methane concentration accurately without the need for further calibration within the range expected in a sewer.

The limit of detection was calculated to be 0.023% vol (i.e. 230 ppm) and 0.110% vol (i.e. 1,100 ppm) for Sensor I and Sensor II, respectively. The calculated relative standard deviations (RSD) in the 20 tests at a methane concentration of 1.35% vol were 2.24% and 1.44% for Sensor I and Sensor II, respectively. Both are smaller than 2.50%, implying good reproducibility.

### Short-term effects of humidity and temperature on sensor performance

**Figure 2:**
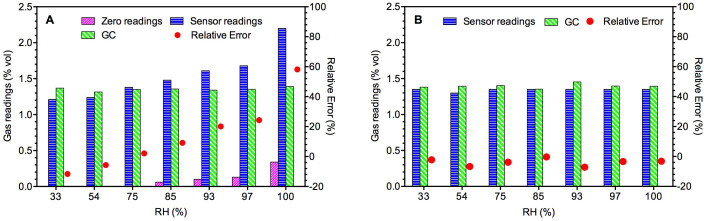
Short-term effect of humidity on Sensor I (A) and Sensor II (B) readings. Zero readings are sensor readings in the absence of methane.

The sensor and GC readings for samples under different laboratory humidity levels are presented in Fig. 2A and Fig. 2B for Sensor I and Sensor II, respectively. Sensor I is significantly affected by humidity. As the sensor was factory calibrated at 40–60% RH, the readings below this level of RH were lower than the actual level measured with GC. On the other hand, the relative error ((Sensor reading – GC reading)/GC reading) increased significantly from 2.1% to 58.1% with the increase in humidity beyond 70% RH. Also, it should be noted that the zero reading (sensor readings in the absence of methane) increased with increased humidity.

On the other hand, the Sensor II readings were relatively stable within the RH range of 33–100% (Fig. 2B). There is no systematic trend in the relative errors, with a mean value of −3.82% and a standard deviation of 2.42%. Also, there was no zero reading drift during the tests (data not shown). Two 4-point calibrations of the sensor were further performed at 85% and 97% RH, respectively. Fig. S1 shows excellent linearity in both cases (R^2^ > 0.99), with slopes and intercept values close to those at 54% RH (Fig. 1). These results clearly indicate that the Sensor II performance is not affected by humidity and the device can provide credible methane readings under a wide range of humidity.

**Figure 3:**
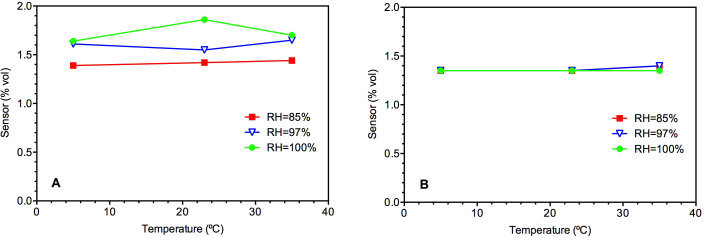
Short-term effect of temperature on Sensor I (A) and Sensor II (B) readings.

Temperature in the range of 5–35°C had negligible impact on the Sensor II readings in the RH range of 85–100% (Fig. 3B). No zero reading drift was observed even under extreme temperature and humidity conditions (i.e. under 100% RH and 5 or 35°C) (data not shown). In comparison, Sensor I showed some variation in its readings when the temperature was varied (Fig. 3A). The variation was relatively small (<12%) but without an obvious pattern.

### Long-term stability under various humidity conditions

**Figure 4:**
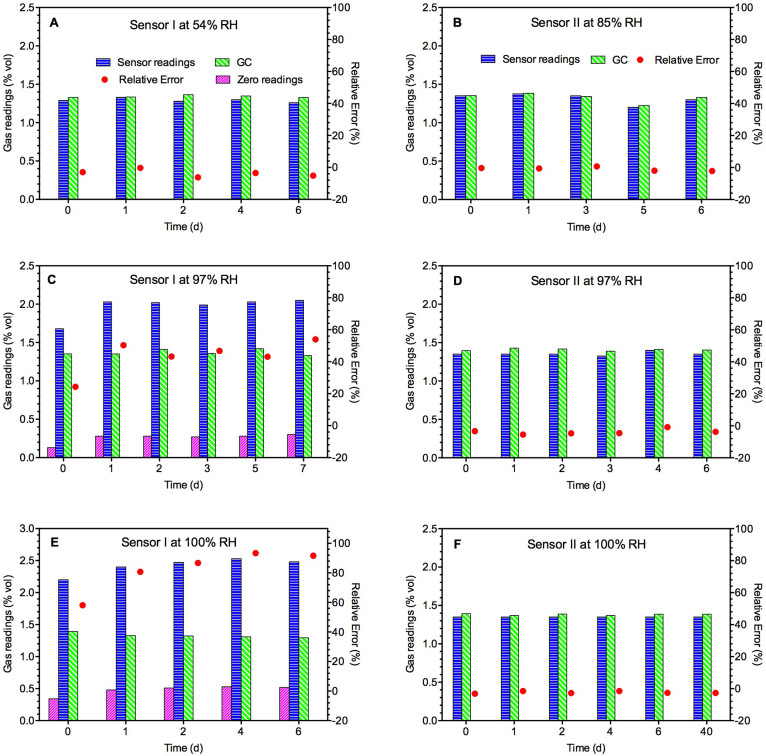
Performance of Sensor I (A, C and E) and Sensor II (B, D and F) during long-term exposure to various levels of RH. Zero readings are sensor readings in the absence of methane.

In this test, Sensor II showed no zero reading drift when used to measure methane concentration of normal air (without methane gas injection) regardless of the humidity level and time. The sensor readings were found to be close to the GC readings at all the RH levels tested (85%, 97% and 100%) (Fig. 4B, D and F). Even after being exposed for 40 days at a condensing condition, the sensor's reading was still stable and close to the GC reading.

For Sensor I, the relative error was small and consistent during a 6-day test at 54% RH (Fig. 4A). In this case, there was no zero reading drift. However, at higher RH levels of 97% and 100%, the sensor reading increased significantly after 1 day, and then remained at a relatively constant level, which was 47% and 88% higher than the GC readings (Fig. 4C and 4E), resulting in large relative errors in measurement. The sensor showed a substantial zero reading drift of 0.28% vol and 0.51% vol, respectively.

### Sensor I performance at increased surface temperature

**Figure 5:**
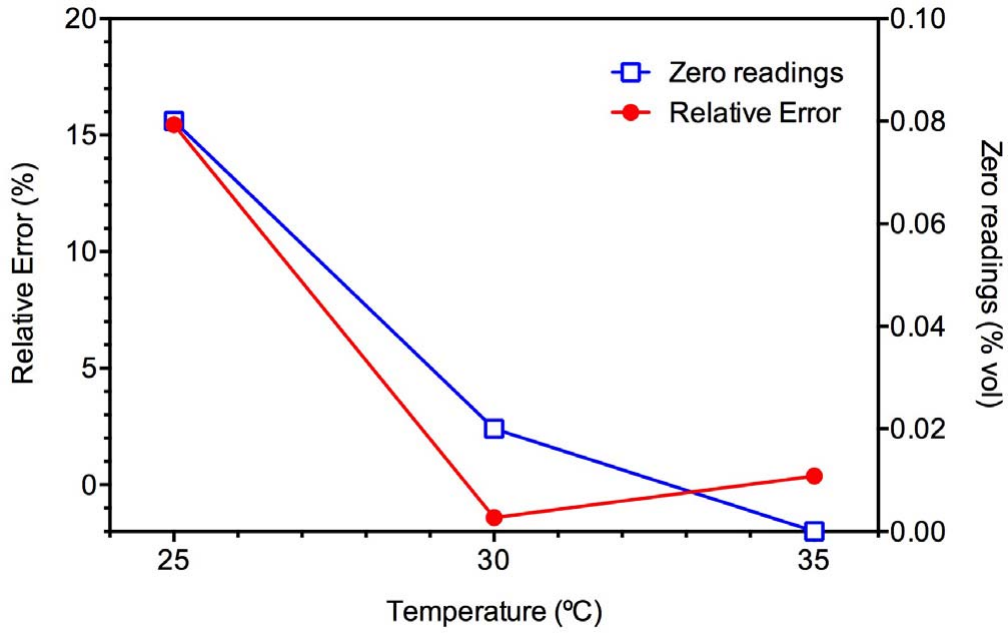
Effect of surface temperature of Sensor I on the relatively measurement error and zero drifting at 93% RH.

We hypothesized that the different sensitivity of the two sensors to humidity could be related to the different surface temperatures of the sensors during their operation. With an external power supply, Sensor II consumes a large amount of power (3.6–6.25 W, 150–250 mA at 24 VDC), and the heat produced led to a surface temperature around 35°C. This likely eliminates the impact of water vapor. In comparison, operated with a Ni-MH integrated battery pack, portable Sensor I was designed to have a lower power consumption and has a surface temperature close to the environment (about 25°C on the sensor surface as room temperature). Further laboratory experiments (Fig. 5) showed with the surface temperature increased from 25°C to 30°C and 35°C (using a heating chip), the relative error of Sensor I at 93% RH reduced from about 15% to −1.4% and 0.4%, respectively, comparable to those obtained with Sensor II. Similarly, the zero reading decreased from 0.08% vol at 25°C to 0.02% vol and zero at 30°C and 35°C, respectively. The relative error of Sensor I stayed at a negligible level (<0.6%) in a further test, during which the sensor was exposed to 93% RH for two days (Fig. S2), contrasting the results shown in Fig. 5. These results clearly indicate that increasing surface temperature of the sensor is effective to eliminate the effect of humidity on Sensor I readings.

### Field application and evaluation of sensors

**Figure 6:**
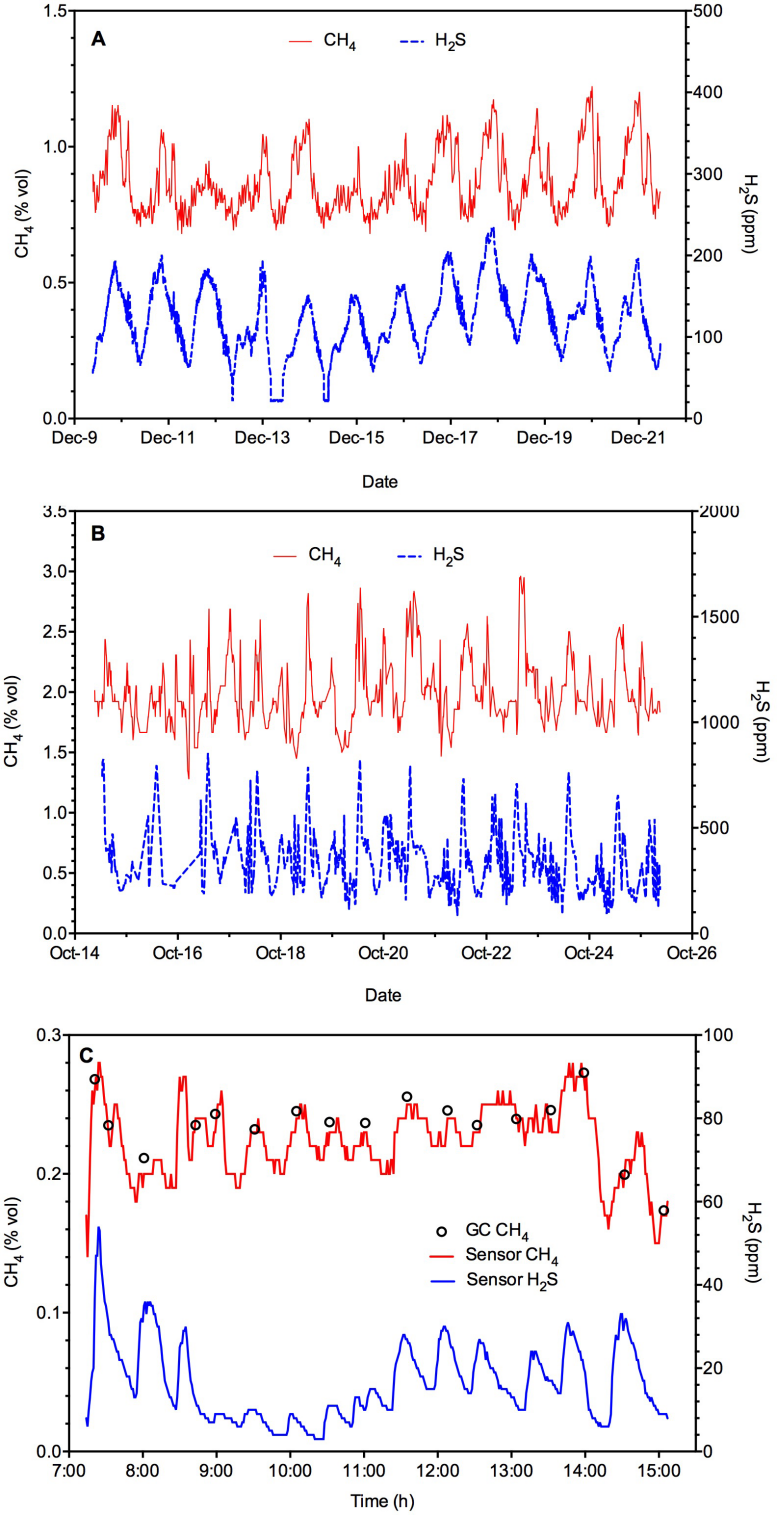
CH_4_ and H_2_S profiles at Manhole A (A), Manhole B (B) and at pumping station C (C).

Fig. 6A and 6B presents the field study results acquired with Sensor II. Temperature and humidity levels were relatively stable and these averaged to 28.3 ± 0.3°C and 97.9 ± 0.4% RH for Manhole A, and 24.2 ± 0.3°C and 87.3 ± 3.6% RH for Manhole B during the entire measurement campaign (Fig. S3). The CH_4_ concentrations varied between 0.7% to 1.2% vol with an average concentration of 0.9% vol in Manhole A. In comparison, it fluctuated from 1.5% to 2.9% with an average concentration of 2.0% vol in Manhole B. In both cases, the CH_4_ profile displayed a clear diurnal pattern. Manhole B had higher concentrations than Manhole A, possibly because it receives discharge from a pressure sewer dominated by anaerobic conditions. In comparison, Manhole A receives discharge from a gravity sewer, in which the transfer of CH_4_ from liquid to gas is presumably an on-going process due to the presence of liquid and gas interface throughout the sewer line. The CH_4_ profile in Manhole B also displayed more frequent spikes, possibly related to the intermittent pump operation feeding the pressure main. In both cases, the CH_4_ diurnal pattern is very similar to that displayed by the H_2_S profiles, which varied between 50 to 200 ppm for Manhole A and 100 to 800 ppm for Manhole B, respectively. Our previous research has shown that CH_4_ and H_2_S are simultaneously produced in sewers^19^. The almost identical pattern in the two profiles suggests that both the CH_4_ and H_2_S sensors are capturing the dynamics correctly.

Fig. 6C shows the field data obtained by Sensor I with humidity control device (see Field application in the Methods section) from the pumping station C. Several gas samples were also manually taken and measured with GC to validate the sensor results. The average temperature and humidity during the measurement campaign at this site were 28.3°C and 90.3% RH, respectively. The sensor readings (with humidity control) have an excellent fit with the GC data. Also, it should be noted that the CH_4_ profile displayed a similar trend to that of the H_2_S data. The methane concentration was not as high as that measured at the other two sites (Fig. 6A and 6B). Similarly, the H_2_S concentration at this site was also substantially lower than that at the other two sites (Fig. 6A and 6B). The reason could be that this pumping station receives sewage from three small upstream gravity sewers, and methane and H_2_S emissions should have been an on-going process in the upstream sewer pipes.

## Discussion

While many sensors are available for on-line monitoring of CH_4_ concentrations, their application in sewer environment has not been investigated to date. A key feature of sewer air is its high humidity with RH typically in the range of 80–100%, which could potentially interfere with CH_4_ measurement. One of the two sensors displayed excellent robustness towards high humidity and its variation. While the other was found to be sensitive to humidity, solutions were developed and demonstrated in this work to resolve issue. The robustness against humidity could be achieved either by elevating the surface temperature of the sensor or by developing a device that reduces humidity to RH levels in the range of 40–60%. The sensors are insensitive to temperature variations. Excellent performance was obtained with both sensors in the short- and long-term tests in both laboratory and field.

Both sensors exhibited excellent linearity in the calibration studies with R^2^ values above 0.999, slopes close to 1 and intercepts close to 0. These results imply that the sensors can be applied with factory calibration.

The two sensors had a detection limit of 0.023% vol (Sensor I) and 0.110% vol (Sensor II), respectively. The three field measurement campaigns showed that these limits are much lower than the values measured. However, it should be noted that the CH_4_ concentration data reported in the US study^10^ are below the detection limit of Sensor II. In this case, the sensors will not be able to provide accurate measurement. However, the CH_4_ emission in this case is expected to be low, and its accurate quantification would not be critical.

After comprehensive laboratory evaluation, both sensors were applied to actual sewer conditions. This is to our knowledge the first time that performances of different online methane gas sensors for measuring sewer gas were rigorously and systematically evaluated in sewer air. The results revealed a substantial presence of CH_4_ in sewer air (3–4 orders of magnitude higher that in atmosphere), supporting the conclusions drawn in several recent studies that sewers are a source of methane and CH_4_ is produced from sewers in significant quantities^7^,^8^,^9^. The CH_4_ profile displayed a clear diurnal pattern, which is likely caused by the diurnal variation of HRT in sewer networks^20^. In addition, the intermittent pump operation brings further variations to the CH_4_ profile. These results further confirmed that the CH_4_ concentration in sewer air is highly dynamic and cannot be accurately quantified through infrequent (i.e. hourly) manual sampling. The IR-based CH_4_ sensors, in conjunction with gas flow meters, provide powerful tools for quantifying CH_4_ emissions from sewers. The continuous measurement also provides information-rich data to the calibration of mathematical models for the prediction of CH_4_ emissions from sewers^7^,^19^.

In summary, this study revealed that IR sensors are suitable for measuring gas phase CH_4_ concentrations in sewers with concentrations above the detection limits, which may not be the case for all sewers as stated earlier. Sensor I, operated with battery, is suitable for short-term preliminary quantification of methane concentration before intensive measurement. In contrast, Sensor II, operated with an external power supply, is more suitable for long-term quantification to identify the weekly, monthly and seasonally variations in methane emissions.

## Methods

### Sensors

Two IR CH_4 _sensors, i.e. one portable gas detector (Sensor I) and one fixed gas detector (Sensor II), were selected for both the laboratory testing and in-situ evaluation. The key specifications of the two sensors are summarized in Table 1.

Sensor I is designed as a personal safety device for petrochemical industry and underground mining, but is also claimed to be applicable in the wastewater industry for CH_4_ measurement at sewerage pumping stations, sewer manholes, inside of sewer collection lines, drains and pits. The sensor is claimed to be corrosion-resistant. According to the sensor manual, temperature and relative humidity can affect the sensor performance. As it is battery-operated, it can only be applied for about 24 hours in each application. It is able to log data for this period at a frequency of up to 1 s^−1^.

Sensor II has similar specifications except that it has a resolution of 0.05% vol, which is five times that of Sensor I. It requires an external power supply and hence can be operated for a longer period of time.

### A laboratory setup for sensor testing

The two gas phase methane sensors were evaluated in the laboratory for linearity, detection limit, reproducibility and effects of environmental conditions such as humidity and temperature.

**Figure 7:**
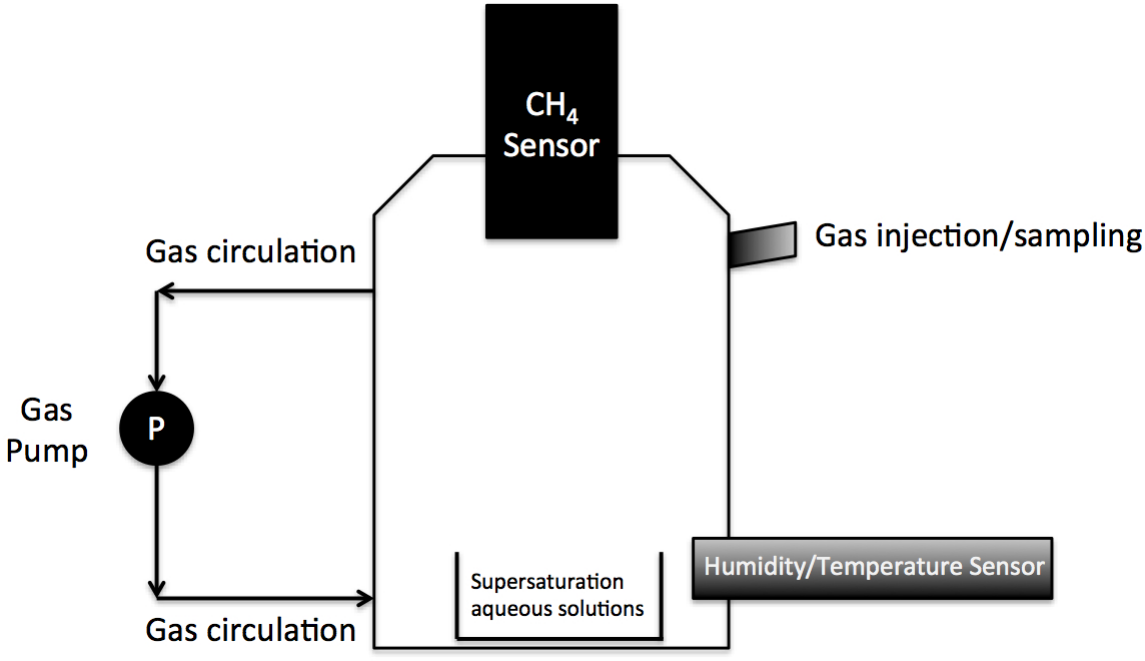
Schematic diagram of the experimental setup.

Fig. 7 shows the experimental setup for the laboratory testing of the sensors' performance. The gas-phase methane sensor was placed at the top of an airtight chamber (700 mL). Relative humidity (RH) in the chamber was controlled at an intended level by adding supersaturated aqueous salt solutions (30 mL) of MgCl_2_, Mg(NO_3_)_2_, NaCl, KCl, KNO_3_, K_2_SO_4_ into a container inside the chamber, which are estimated to yield approximately 33, 54, 75, 85, 93 and 97% RH at room temperature (20°C), respectively^21^. Also, the RH levels thus generated are quite stable in the temperature range of 5–35°C^22^. 100% RH was acquired by adding water only. Three different temperatures were created during the testings by placing the chamber in 3 different temperature-controlled rooms, namely the cold room (5 ± 1°C), an air-conditioned laboratory (23 ± 1°C), and an anaerobic incubator (35 ± 1°C). An air recirculation pump (Xinweicheng, FML201.5, flow rate 1.5 L/min) was used to ensure complete mixing in the gas chamber during the tests. Also, a humidity/temperature sensor (KIMO, HST-D) was placed inside the chamber to monitor the humidity and temperature continuously. The sampling port on the top of the chamber was used to inject known amounts of the methane gas into the chamber, and to take gas samples for GC analysis. The chamber was fully sealed during the tests.

### Experimental design

#### Experiments to determine linearity, detection limits and reproducibility

The sensors' linearity of response, limits of detection and reproducibility were tested. During the tests, different volumes of 90% methane gas (also containing CO_2_ and N_2_ at 5% each) were injected to the measurement chamber to achieve selected methane concentrations in the gas, giving rise to theoretical concentrations in the range of 0.1–5% vol. The tests allowed the evaluation of linearity and limits of detection. All these tests were done under a room temperature of 23 ± 1°C and a humidity level of 54% RH, under which factory calibration of both sensors was carried out. In order to check whether the sensor gave credible readings, gas samples were taken from the reactor in each test after well-mixed conditions were established and transferred to pre-evacuated Exetainers (Labco, Wycombe, UK). The gas samples were subsequently analyzed with Gas Chromatograph (GC) for their CH_4_ concentrations. The GC results thus obtained were compared with the sensor readings to evaluate the sensor's performance in terms of the linearity (R^2^ value of the fitting curve) and limit of detection (defined as the lowest concentration of a substrate that can be determined by a certain method with 99% confidence that the concentration is higher than zero). In this study, the limit of detection was calculated by using the formula as 3.3 σ/S^23^, where σ is the standard deviation of the response relatively to the GC-measured values, and S is the slope of the fitting curve. In addition, the reproducibility of measurement using the two sensors was assessed by repetitive measurement of 20 individual methane samples with the theoretical methane concentration of 1.35% vol under the same operating conditions as described above.

#### Short-term tests on the effects of humidity and temperature

Before each short-term test, the intended RH and temperature levels were maintained in the chamber for 2–4 hours to achieve stable conditions in the chamber. Then the cap of the chamber was opened for a very short time (approximately 10 s) so that the sensor could be quickly put into the chamber and the sensor was left for 0.5–1 h, which ensured stable RH reading. 10 mL of a gas containing CH_4_ at 90% was then injected to the chamber. When the sensor reading became stable (~3 mins), a gas sample was taken from the reactor and transferred to a Labco Exetainer for further GC analysis. For the study of the effect of humidity on the sensor performance, the temperature was at 23 ± 1°C. For the study of effect of temperature on the sensor performance, the RH levels were 85, 97 and 100% for each of the 3 different temperature conditions, namely 5 ± 1°C, 23 ± 1°C, and 35 ± 1°C.

The tests above revealed that Sensor II was robust against short-term humidity change. Therefore, two 4-point calibrations of Sensor II were further performed at 85% and 97% RH (23 ± 1°C), respectively.

#### Long-term performance tests

Continuous measurement of methane in the field under humid conditions was expected to have some interference due to accumulation of water vapor near the sensor optical path or condensation of water vapor. The long-term stability tests were therefore conducted in laboratory prior to field tests to examine their long-term performance by continuously placing the sensor in a specific humid condition for several days, and injecting methane gas at regular interval (every 1–2 days). The sensor performance was monitored by comparing sensor readings with GC data after the gas injection. The experiments allowed to obtain two different observations: (1) apparent sensor reading under zero methane concentration - before methane gas is injected; and (2) sensor reading for a known gas phase methane concentration - after methane gas is injected. Since Sensor II was robust against long-term humidity exposure, a further test after being exposed for 40 days at a condensing condition was conducted. Details of the testing procedures are summarised below.

##### Sensor II

The following experimental procedure was applied every 24 hours:

1. The sensor was continuously placed under given humidity and temperature conditions.
2. 10 mL of 90% CH_4_ gas was injected to the chamber. When the sensor reading became stable (~3 mins), a gas sample was taken from the chamber for further GC analysis.
3. The valve on the chamber was opened and the chamber was flushed with air for 5–10 mins at a flow rate of 1.5 L/min to strip CH_4_ from the chamber (the CH_4_ readings by the IR sensor became zero). During this period, the humidity also changed towards that in the ambient condition.
4. The valve was closed. The desired humidity and temperature conditions were reestablished after 1–2 hours.
5. After about 24 hours, Steps 2–4 were repeated for additional tests.

##### Sensor I

This sensor is powered with a battery with a normal measurement cycle of ~24 hr. To be able to examine the sensor performance over several days under a constant condition, different from the procedure applied to Sensor II, Sensor I was switched off between measurements to prolong the battery life. During each measurement, the sensor was turned on. Then Steps 2–5 were followed except that the sensor was turned off after Step 4.

### Increasing the surface temperature on Sensor I

The tests above revealed that Sensor I was sensitive to humidity while Sensor II was robust against humidity. To understand the mechanism involved and also to improve the robustness of Sensor I towards humidity variation, we modified Sensor I by adhering a heating chip (1 cm × 1 cm) at the sensor probe surface to increase the probe surface temperature from 25 to 35°C, a temperature measured at the surface of Sensor II probe (the Sensor II surface temperature was at this level despite the variation of ambient temperatures, due to its higher power consumption). The Sensor I surface temperature was controlled by changing the voltage on the heating chip. The experiment was conducted at 93% RH condition with a procedure as described for the short-term tests.

### Field application

To verify performance of the sensors under in-situ conditions, Sensor II was installed at the headspace of two discharge manholes in two sewer lines. Manhole A receives discharge from a large gravity sewer with a pipe diameter of 1800 mm. The daily flow is about 17,000 m^3^/d. Manhole B receives discharge from a 15-km long pressure sewer with a pipe diameter of 900 mm. The daily flow is about 11,000 m^3^/d. A temperature sensor (SL-H_2_S-200, Odalog), a humidity sensor (HMP60, Vaisala) and a hydrogen sulfide sensor (SL-H_2_S-200, Odalog) were installed along with Sensor II. The measurement campaign at each site lasted for 12 days, with data logged by all sensors collected at the end.

Due to the short lifetime of the battery (~24 hrs), Sensor I was tested for 8 hrs (daytime) at a local Pumping Station (Site C). As the laboratory evaluation revealed that high humidity affected its performance, Sensor I was installed at the headspace of the pumping station with a device similar to that shown in Fig. 7. A gas pump continuously transported the gas from the sewer headspace to the chamber and then back to the sewer. A chiller (Resun CL85 Nano Chiller) was set in the gas line feeding the chamber to maintain the humidity in the chamber at the desired level of 50–60% RH. This pumping station receives wastewater at a daily flow of 2000 m^3^/d from three upstream gravity sewers, with lengths ranging from 1.0 to 3.0 km and diameters from 225 to 375 mm. Gas samples were manually taken from the chamber, at an interval of about 30 mins during the 8–hr measurement period, using the protocol described in the next section. The samples were analyzed for CH_4_ concentrations with GC, to verify the data measured by the sensor.

### Off-line chemical analysis

For the analysis of gas methane concentration, 25 mL of gas sample was collected using a syringe through the sampling port installed on the chamber and then immediately injected into a 12 mL pre-vacuumed Labco Exetainer^24^. The methane gas in the Exetainer was measured by GC (Agilent 7890A) equipped with a flame ionization detector (FID) within 1–2 weeks after sample collection.

## Author Contributions

Y.L., K.S. and Z.Y. wrote the main manuscript in consultation with all other authors; Y.L., K.S., S.M. and Z.Y. developed the experimental plan and performed data analysis; Y.L. conducted all laboratory experiments and field studies, with I.J. and T.E. contributing to field studies and the associated data analysis; Y.L. and K.S. prepared all figures; All authors reviewed the manuscript.

## Supplementary Material

Supplementary Information Supplementary material for On-line monitoring of methane in sewer air

## Figures and Tables

**Table 1:** Specifications of two IR methane sensors

	Sensor I	Sensor II
Name	OdaLog 7000 IR	GasTech S-Guard IR
Detection range	0–5.00% vol	0–5.00% vol
Resolution	0.01% vol	0.05% vol
T90 Response time^a^	<35 seconds	<10 seconds
Temperature range	−20 to 40°C	−20 to 50°C
Relative humidity range	0 to 95% non-condensing	5 to 99% non-condensing
Operation power	Battery	24 V DC

^a^T90 response time is the time required for 90% change in the sensor signal.
