# Supplementary material for: On-line monitoring of methane in sewer air
Source: Sci Rep. 2014 Oct 16;4:6637. doi: 10.1038/srep06637 (PMC4198865; doi:10.1038/srep06637)
Supplement: Supplementary Information — Supplementary material for On-line monitoring of methane in sewer air [file srep06637-s1.pdf]

Supplementary material for

On-line monitoring of methane in sewer air

Yiwen Liu[a], Keshab R Sharma[a], Sudhir Murthy[b], Ian Johnson[c], Ted Evans[d] and Zhiguo Yuan[a,*]

[a] Advanced Water Management Centre, The University of Queensland, QLD, Australia

[b] District of Columbia Water and Sewer Authority, Washington DC 20032, USA

[c] Gold Coast City Council, QLD, Australia

[d] Water Corporation, WA, Australia

**Corresponding author*: Zhiguo Yuan, Tel.: +61 7 3365 4374; fax: +61 7 3365 4726.

E-mail address: z.yuan@awmc.uq.edu.au

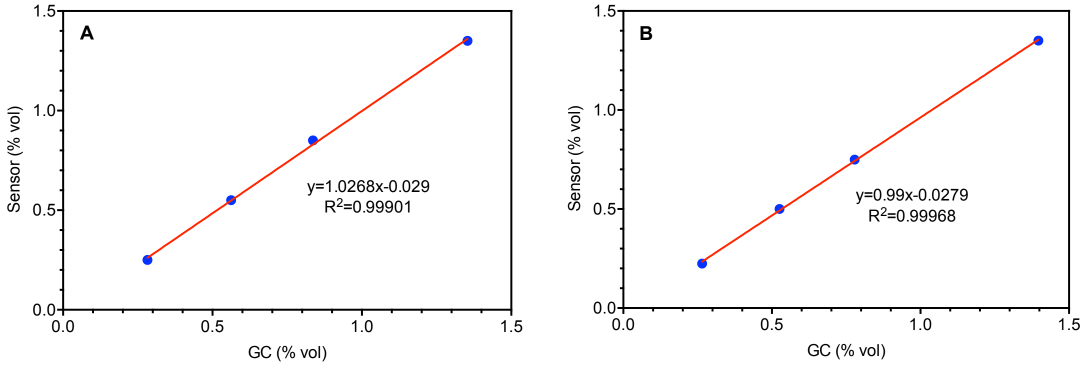

Figure S1 Calibration of Sensor II at (A) 85% RH and (B) 97% RH, both at room temperature (23±1 ºC)

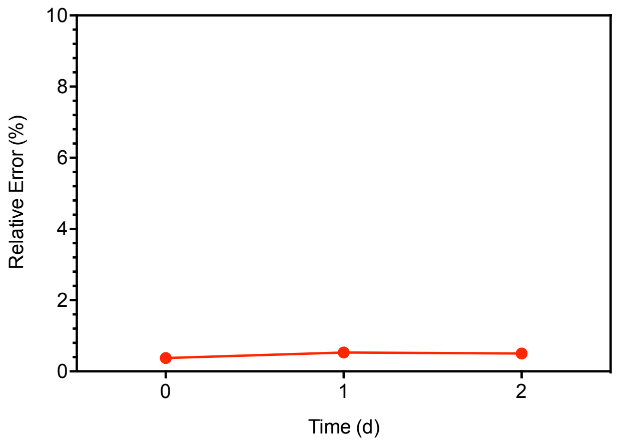

Figure S2 Performance of Sensor I at a surface temperature of 35 ºC during long-term exposure to 93% RH

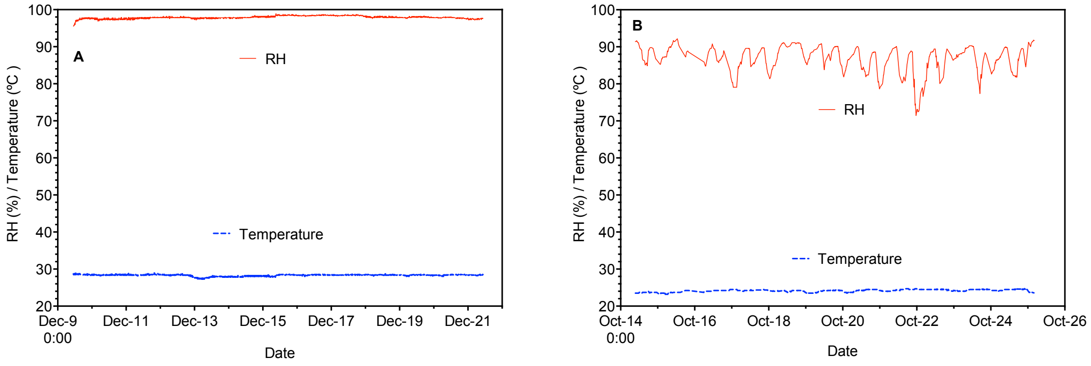

Figure S3 RH and temperature profiles at Manhole A (A) and Manhole B (B).
